# Angiogenesis in ossification of the posterior longitudinal ligament: progress from mechanism to targeted intervention

**DOI:** 10.3389/fcell.2026.1707176

**Published:** 2026-03-04

**Authors:** Xiaoyu Liu, Xiaomin Wang, Kangyi Hu, Haonan Wen, Lu Liu, Haoxing Li, Zhixin Che, Ting Song, Jinquan Lai, Min Song, Yongjia Song

**Affiliations:** 1 Clinical College of Chinese Medicine, Gansu University of Chinese Medicine, Lanzhou, China; 2 Spinal Orthopedics, Shenzhen Luohu Hospital of Traditional Chinese Medicine, Shenzhen, China

**Keywords:** angiogenesis, angiogenesis-osteogenesis coupling, ossification of the posterior longitudinal ligament, signaling pathway, targeted intervention

## Abstract

Ossification of the posterior longitudinal ligament (OPLL) is a degenerative spinal disorder characterized by heterotopic ossification of ligamentous tissue. Its pathogenesis is multifactorial and complex, involving genetic susceptibility, chronic inflammation, mechanical stress, and metabolic dysregulation. In recent years, accumulating evidence has demonstrated that angiogenesis not only supplies essential nutrients and metabolic support to ossified ligament regions but also actively regulates the differentiation of mesenchymal stem cells toward osteogenic and chondrogenic lineages through specific molecular signaling pathways, thereby promoting ectopic bone formation. Focusing on angiogenesis as a central theme, this review systematically summarizes the mechanisms by which key molecules, including LOXL2, Sema3A, integrin αVβ3, ANGPT2, IL-6, TGF-β, the *ACE* D/D polymorphism, and YAP, mediate the coupling of angiogenesis and osteogenesis in OPLL. Furthermore, we propose angiogenesis-targeted strategies as a potential therapeutic avenue for OPLL, aiming to provide new theoretical insights and directions for both basic research and clinical intervention.

## 1 Introduction

Ossification of the posterior longitudinal ligament (OPLL) is a relatively rare pathological process characterized by progressive lamellar bone deposition within ligamentous tissue. Continued ossification can result in spinal canal stenosis, spinal cord compression, and subsequent neurological dysfunction, making OPLL a major etiological factor in cervical spondylotic myelopathy (CSM) (Boody et al., 2019; Head et al., 2019). Epidemiologically, OPLL exhibits marked racial and sex-related disparities, with a significantly higher prevalence in East Asian populations and an approximate male-to-female ratio of 2:1 (Choi et al., 2011). In Japan, the prevalence of OPLL ranges from 1.9% to 4.3%, whereas lower rates have been reported in Europe and North America (0.1%–1.7%) (Le et al., 2022). In the Chinese population, the prevalence of OPLL in the cervical, thoracic, and lumbar spine is reported to be 4.1%, 2.25%, and 0.8%, respectively (Liang et al., 2019). Regarding segmental distribution, approximately 92% of OPLL cases involve the cervical spine, most commonly affecting the C4-C6 levels, while involvement of the upper cervical spine (C1-C2) is rare. Thoracic and lumbar OPLL account for the remaining ∼8% of cases, with a predilection for the T1-T4 segments (Kawaguchi et al., 2018; Pham et al., 2011). Notably, thoracic OPLL is associated with a higher rate of severe neurological impairment and disability compared with cervical involvement (Kaliya-Perumal et al., 2018).

OPLL has not yet been fully elucidated; however, its early histopathological features closely resemble those of diffuse idiopathic skeletal hyperostosis (DISH) (Mader et al., 2017; Resnick et al., 1978). Genetic studies have demonstrated a strong heritable component in OPLL. Genome-wide association studies (GWAS) have identified multiple susceptibility loci, including *RSPO2, BMP9, TGFBR2, COL6A1, IL17RC*, and *TMEM135*, many of which are closely associated with the Wnt/β-catenin and TGF-β signaling pathways. These findings suggest that OPLL may arise from dysregulated interactions between bone metabolism and immune regulation (Jekarl et al., 2013; Koike et al., 2023; Nakajima et al., 2016; Ren et al., 2012; Wang P. et al., 2018). Epidemiological and Mendelian randomization studies further indicate a causal relationship between increased body mass index (BMI), elevated bone mineral density (BMD), and the progression of OPLL (Endo et al., 2020; Koike et al., 2023). In terms of hormonal metabolism, elevated serum leptin levels and imbalances in male sex hormones have also been implicated in OPLL pathogenesis (Feng et al., 2018; Li et al., 2007). Compared with primary OPLL, secondary forms are more commonly associated with endocrine disorders, including X-linked hypophosphatemia rickets, hypoparathyroidism, and acromegaly (Ikegawa, 2014). Despite these advances, which provide important insights into the multifactorial nature of OPLL, the core pathogenic mechanisms underlying disease initiation and progression remain incompletely understood.

Notably, angiogenesis recognized as a central biological process in bone development and remodeling (Stegen et al., 2015) has emerged in recent years as a major focus of OPLL research. Under physiological conditions of bone repair, angiogenesis is tightly coupled with osteogenesis: newly formed vessels not only supply oxygen and nutrients but also regulate the differentiation and maturation of osteoprogenitor cells through the secretion of signaling molecules such as vascular endothelial growth factor (VEGF), fibroblast growth factor (FGF), and platelet-derived growth factor (PDGF) (Hankenson et al., 2011; Peng et al., 2020). Accumulating evidence indicates that this angiogenesis-osteogenesis coupling is aberrantly activated in OPLL. Ligament tissues from patients with OPLL exhibit a marked increase in CD31-positive vessels (Geng et al., 2024), accompanied by upregulated expression of osteogenic factors (Sato et al., 2007), suggesting an active role for vascular-osteogenic interactions in disease progression. Using combined serum and tissue cytokine profiling, Fay (Fay et al., 2023) further demonstrated significantly elevated expression of angiopoietins (ANGPTs) in OPLL lesions. In parallel, osteoclastogenesis inhibitory factor (OPG), a molecule with both pro-angiogenic and anti-bone resorption properties was highly expressed in OPLL tissues, with tissue levels approximately sixfold higher than those detected in serum. Immunohistochemical analyses revealed that OPG was predominantly localized to vessel-like structures, whereas ANGPTs and osteopontin (OPN) were broadly distributed in perivascular regions, spatially linking angiogenesis to the osteogenic microenvironment. Consistent with these findings, hematoxylin-eosin and Masson trichrome staining demonstrated the presence of neovascular-like structures and elastic fiber disruption within ossified regions. Collectively, these observations provide compelling evidence that active angiogenesis is a defining feature of OPLL lesions. Beyond supplying metabolic support for ectopic bone formation, angiogenesis directly drives OPLL progression by reshaping the local microenvironment. Accordingly, this review systematically examines the role of angiogenesis in the pathogenesis of OPLL and explores the therapeutic potential of angiogenesis-targeted strategies, with the aim of offering new insights for both basic research and clinical intervention.

## 2 The pathological and cytological characteristics of OPLL

### 2.1 Ossification leading edge

OPLL represents a distinct subtype of heterotopic ossification of tendons and ligaments (HOTL) (Zhang et al., 2020). It can be conceptualized as an aberrant repair process initiated by chronic inflammation and driven predominantly by endochondral ossification as the central pathological program (Sakai et al., 2022; Zhang et al., 2020). Histopathological analyses have demonstrated that the active ossification front in OPLL exhibits a well-organized, three-zone architecture, consisting of a fibrocartilage proliferation zone, a zone of chondrocyte hypertrophy and calcification, and a bone remodeling zone (Ikuta et al., 2023; Ono et al., 1999).

### 2.2 The origin and differentiation of MCSs in OPLL

Regarding the cellular origin of MSCs in heterotopic ossification of tendons and ligaments (HOTL), current evidence predominantly points to tendon stem/progenitor cells (TSPCs) (Dai et al., 2020; Feng et al., 2020), rather than bone marrow-derived cells. TSPCs possess key stem cell properties, including self-renewal, clonogenicity, and multilineage differentiation potential (Bi et al., 2007). Advances in single-cell transcriptomic analyses have identified multiple TSPC subpopulations characterized by the expression of markers such as CD26 (Chen et al., 2025), CD55, and CD248 (Tsutsumi et al., 2026). Notably, a CD26-positive peritendinous TSPC subset can be activated under conditions of abnormal mechanical loading, hypoxia, or inflammation and has been shown to directly drive heterotopic ossification (Chen et al., 2025). In specific disease contexts, TSPCs are often operationally defined as ligament-derived stem cells (LDSCs) or tendon-derived stem cells (TDSCs). Spinal ligament-derived cells (SLDCs) isolated from both OPLL patients and healthy controls exhibit canonical MSC phenotypes; however, SLDCs from OPLL patients display significantly enhanced proliferative capacity and osteogenic differentiation potential (Harada et al., 2014; Kim et al., 2023). These findings suggest that SLDCs represent a core pathogenic cell population in OPLL. Moreover, the elevated levels of oxidative stress observed in OPLL-derived SLDCs further support a close association between OPLL pathogenesis and a hypoxic microenvironment within affected lesions (Kim et al., 2023). Beyond SLDCs, several tissue-resident progenitor populations including TDSCs (Asai et al., 2014), LDSCs (Lee et al., 2017), and bone-cartilage stromal progenitors (BCSPs: defined as AlphaV^+^/CD105^+^/Tie2^−^/CD45^−^/Thy1^−^/6C3^−^) (Agarwal et al., 2016b) have been identified as important sources of chondrogenic precursors and are implicated in fibrocartilage formation and osteochondral remodeling. Although some studies suggest that endothelial cells may acquire MSC-like phenotypes through endothelial-to-mesenchymal transition (EndMT) and thereby contribute to heterotopic ossification (Agarwal et al., 2016a; Sun et al., 2016), other evidence indicates that endothelial cells are not a major cellular source in trauma-induced heterotopic ossification (Eisner et al., 2020). Additional candidate cell populations, including perivascular cells, circulating progenitors, and endoneurial cells, have also been proposed; however, direct experimental evidence supporting their roles remains limited (Zhang et al., 2020). In summary, current consensus favors TSPCs as the principal pathogenic cell population driving OPLL and HOTL, while other cell types may contribute to heterotopic ossification in context-dependent or supportive roles. Further investigation is required to fully elucidate the relative contributions and interactions of these cellular sources.

### 2.3 Endochondral ossification program (cascade regulation of MSCs-chondrocytes - osteoblasts)

OPLL follows an endochondral ossification program and is governed by a tightly regulated cascade involving MSCs, chondrocytes, and osteoblasts. At the initial fibrocartilage proliferation zone, pathological stimuli such as hypoxia and inflammation drive the differentiation of MSCs into chondrocytes (Lu et al., 2025; Saito et al., 2023; Sugita et al., 2020), leading to the formation of a cartilage-like extracellular matrix rich in type II collagen and proteoglycans (Sato et al., 2007). At this stage, the hypoxic microenvironment stabilizes hypoxia-inducible factor-1α (HIF-1α), thereby initiating a pro-angiogenic program that establishes the molecular foundation for subsequent vascular invasion. *In vitro* studies have demonstrated that hypoxia mimicked by CoCl_2_ treatment markedly upregulates HIF-1α expression in ligament-derived cells, along with increased expression of downstream angiogenic factors such as VEGFA and PDGF-BB (Lu et al., 2025; Wang et al., 2024). As the lesion progresses into the zone of chondrocyte hypertrophy and calcification, chondrocytes undergo hypertrophic differentiation under the influence of signaling molecules such as bone morphogenetic proteins (BMPs), express type X collagen, and subsequently undergo apoptosis, accompanied by matrix mineralization and formation of a calcified cartilage template (Ikuta et al., 2023). During this phase, VEGF secreted by hypertrophic chondrocytes serves as a key guidance cue for vascular invasion (Sato et al., 2007; Yayama et al., 2007), thereby mechanistically coupling angiogenesis with osteogenesis. The ingress of neo vessels facilitates the recruitment of osteoclast precursors and osteoprogenitor cells into the calcified cartilage. Osteoclasts resorb the mineralized cartilage matrix, while osteoblasts deposit woven bone on the residual cartilage scaffold, which is subsequently remodeled into mature lamellar bone. Importantly, neo vessels function not only as conduits for metabolic supply but also as active signaling hubs, as endothelial cells secrete osteogenic factors that further promote ossification (Peng et al., 2020; Wang et al., 2024). Taken together, the pathological essence of OPLL may be conceptualized as an aberrant and sustained coupling of endochondral ossification and pathological angiogenesis within ligamentous tissue, driven by abnormal mechanical loading, inflammation, or hypoxia through activation of the HIF-1α/VEGF signaling axis.

### 2.4 Inflammation-angiogenesis interaction

The interplay between inflammation and angiogenesis represents a critical nexus driving the progression of OPLL. Prolonged abnormal mechanical loading induces sustained hypoxia within ligamentous tissue, thereby triggering inflammatory responses and angiogenic activation. Large numbers of circulating monocytes are recruited to the lesion site and differentiate into macrophages, which, together with MSCs, chondrocytes, and osteoblasts, establish a pro-osteogenic inflammatory microenvironment (Huang et al., 2021; Ikuta et al., 2023; Wu et al., 2025). Clinical evidence indicates that patients with OPLL exhibit significantly elevated peripheral blood monocyte counts, as well as increased levels of IL-1β, TNF-α, and MCP-1; moreover, high-sensitivity C-reactive protein (hs-CRP) levels are markedly higher in mixed-type OPLL than in focal-type disease (Wu et al., 2025). Saito (Saito et al., 2023) further demonstrated that IL-6 is highly expressed in ligament tissue from patients with OPLL and is predominantly localized to MSCs at the ossification front. Through regulation of *Sox9*, *Runx2*, and *Sirt1* expression, IL-6 promotes chondrocyte hypertrophy and apoptosis, thereby contributing to early endochondral ossification. Additional studies have reported extensive accumulation of CD68-positive macrophages in degenerated posterior longitudinal ligament tissue, accompanied by robust secretion of TGF-β1 and the frequent presence of newly formed microvessels within these macrophage-rich regions (Wang et al., 2015). Notably, inflammatory mediators such as IL-6 and TGF-β not only directly drive MSCs or ligament-derived cells toward osteogenic and chondrogenic lineages but also upregulate pro-angiogenic factors, including VEGF and ANGPT2, thereby supporting MSC recruitment, survival, and lineage commitment (Moon et al., 2012; Saito et al., 2023; Tu et al., 2023). Within this context, macrophages serve as a pivotal cellular link between inflammation and angiogenesis (Corliss et al., 2016). Macrophage-derived IL-1β can activate the HIF-1α/VEGF signaling axis, initiating and amplifying pathological angiogenesis (Chen et al., 2022; Chen et al., 2023; Mantsounga et al., 2022; Westra et al., 2010). Moreover, macrophage polarization has emerged as a key determinant of angiogenesis-osteogenesis coupling (Liu et al., 2025; Tu et al., 2023). In trauma-induced heterotopic ossification models, M2-polarized macrophages markedly enhance chondrogenic and osteogenic differentiation of MSCs, whereas macrophage depletion or inhibition of TGF-β signaling significantly attenuates both endochondral ossification and angiogenesis (Tu et al., 2023). Given that OPLL likewise progresses predominantly through an endochondral ossification pathway, it is plausible that early infiltration of pro-inflammatory M1 macrophages amplifies local inflammatory signaling via secretion of IL-1β and TNF-α and activates the HIF-1α/VEGF axis to initiate pathological angiogenesis. Subsequently, polarization toward a reparative M2 phenotype characterized by the secretion of TGF-β, IL-6, VEGF, and ANGPT2 may simultaneously promote MSC and ligament-derived cell differentiation into chondrocytes and osteoblasts, thereby driving endochondral ossification, while sustaining and amplifying local angiogenesis. Through these dual actions, macrophages may establish a central cellular hub linking the inflammatory microenvironment with angiogenesis-osteogenesis coupling, ultimately facilitating ectopic bone formation by providing metabolic support and migratory pathways. Nevertheless, these mechanistic inferences are largely derived from models of heterotopic ossification, and direct experimental evidence specific to OPLL remains limited, warranting further investigation.

**FIGURE 1:**
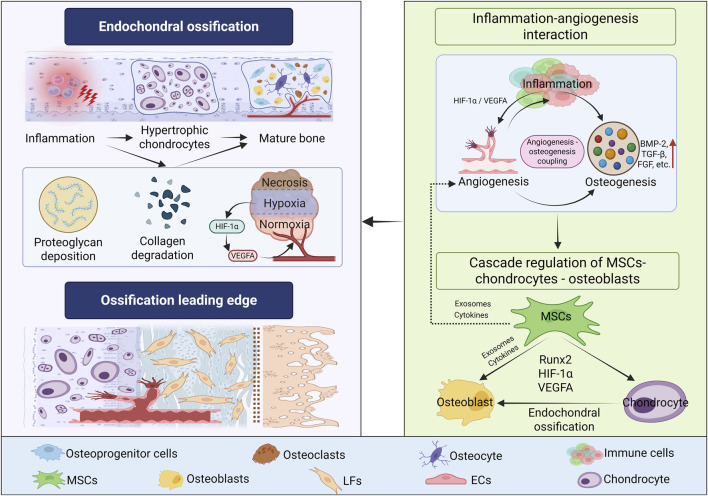
The pathological and cytological characteristics of OPLL. The pathological changes of OPLL often occur secondary to inflammation. Inflammation stimulation mediates chondrocyte differentiation, proteoglycan deposition, collagen matrix degradation, and angiogenesis, forming a transitional zone where chondroid tissue and fibrotic tissue coexist. Eventually, it transforms into an osteogenic phenotype, promoting ectopic bone formation in ligament tissue. Among them, the cascade regulation of MSCs-chondrocytes - osteoblasts and the “inflammation-angiogenesis interaction” are important factors promoting the progression of OPLL. Note: This figure was created by biorender.

In conclusion, based on the re-examination of the histopathological and cytological characteristics of OPLL (Figure 1), future research should focus on analyzing the synergistic network of inflammation, angiogenesis, and osteogenic signals, with the aim of providing a theoretical basis for understanding the pathological mechanism of OPLL and targeted intervention strategies.

## 3 The similarities and differences of the angiogenic-osteogenic coupling mechanism in OPLL and other ectopic ossifications

Angiogenesis-osteogenesis coupling constitutes the central pathological mechanism underlying the progression of heterotopic ossification and can be fundamentally described as an ordered cascade in which hypoxia- and/or inflammation-induced vascular invasion initiates endochondral ossification (Dey et al., 2017; Dilling et al., 2010). OPLL conforms to this canonical paradigm (Sato et al., 2007) and shares several core features with other forms of heterotopic ossification and with fracture healing (Dey et al., 2017; Li et al., 2024; Wang et al., 2024). First, all three processes rely on angiogenesis to supply oxygen, nutrients, and osteokines to the ossifying region, with the HIF-1α/VEGF signaling axis playing a pivotal regulatory role. Second, endochondral ossification represents the predominant mode of bone formation in most contexts. Third, BMP signaling promotes osteogenic differentiation while coordinately regulating angiogenesis in cooperation with VEGF. Despite these shared features, distinct forms of heterotopic ossification differ substantially in their initiating stimuli, dominant signaling pathways, and cellular interactions. OPLL is typically driven by chronic abnormal mechanical stress and persistent inflammation. Angiogenesis in this setting progresses in a localized, sprouting-like manner and engages in specific interactions with ligament-derived cells, resulting in slowly progressive lamellar bone formation that may ultimately lead to spinal canal stenosis (Sato et al., 2007; Tanno et al., 2003; Wang et al., 2024; Wu et al., 2025). In contrast, traumatic heterotopic ossification arises from severe tissue injury, such as fractures, surgical trauma, or burns, and is characterized by an intense early inflammatory response that drives rapid and disorganized vascular expansion. In later stages, osteogenesis becomes predominantly BMP driven, while macrophage polarization and neuropeptide signaling further amplify angiogenesis-osteogenesis coupling, accounting for the clinically aggressive course and high recurrence rate of this condition (Hwang et al., 2022; Ren et al., 2025; Salisbury et al., 2011). Fibrodysplasia ossificans progressiva (FOP), a rare genetic disorder caused by activating mutations in ACVR1, represents a distinct pathological entity. In FOP, constitutive activation of BMP-Smad1/5 signaling and aberrant responsiveness of the mutant receptor to Activin A render even minimal inflammatory stimuli sufficient to trigger multifocal and progressive ossification (Hino et al., 2015; Towler and Shore, 2022). In this context, angiogenesis functions primarily as a downstream effector process, initiated independently of chronic hypoxia or acute tissue injury and instead governed by cell-intrinsic signaling abnormalities. By contrast, fracture healing is a physiological repair process in which angiogenesis-osteogenesis coupling is tightly regulated and appropriately terminated upon completion of tissue reconstruction, thereby avoiding persistent pathological activation (Beamer et al., 2010; Street et al., 2002; Wang et al., 2007). In summary, although angiogenesis-osteogenesis coupling represents a shared pathological foundation across diverse forms of heterotopic ossification, the mechanisms of initiation, regulatory networks, and disease trajectories exhibit pronounced model-specific differences. Systematic delineation of these distinctions will be essential for advancing our understanding of the heterogeneity of heterotopic ossification and for the development of targeted therapeutic strategies.

## 4 Angiogenesis in OPLL

### 4.1 The clinicopathological link between angiogenesis and OPLL

**FIGURE 2:**
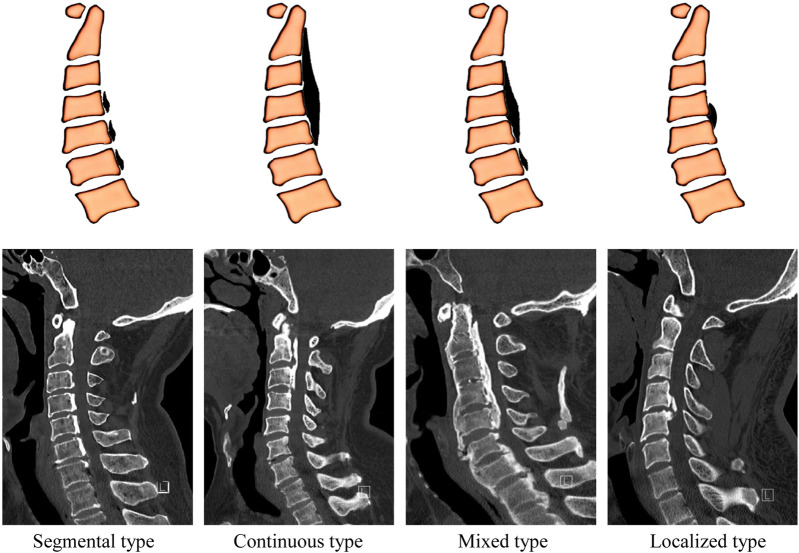
Imaging classification of OPLL. Sagittal CT scan images provided by Dr. Lai Jinquan, Shenzhen Luohu Hospital of Traditional Chinese Medicine.

OPLL is classified into segmental, continuous, mixed and localized types according to its imaging features (Figure 2) (Abiola et al., 2016). Clinical observations have found that the amount of bleeding in the surgical area of patients with continuous OPLL is significantly greater than that of patients with segmental OPLL, and that of patients with mixed OPLL is between the two (Kishiya et al., 2009), suggesting that there are differences in the degree of angiogenesis among different subtypes. Another study showed that the expression levels of osteogenesis-related factors (such as OPN, OPG, TGF-β2) and angiogenic factors (such as ANGPT, VEGF) in the lesion area of OPLL patients were significantly higher than those in the non-OPLL group (Fay et al., 2023). These results suggest that there may be systematic differences in the degree of ossification and vascular distribution among different OPLL subtypes, which have not been fully confirmed yet. Therefore, future in-depth exploration of the heterogeneity of vascular responses in different subtypes is expected to further reveal the pathological mechanism of the coupling of angiogenesis and osteogenesis in OPLL.

### 4.2 Angiogenesis accelerates the pathological process of OPLL

**FIGURE 3:**
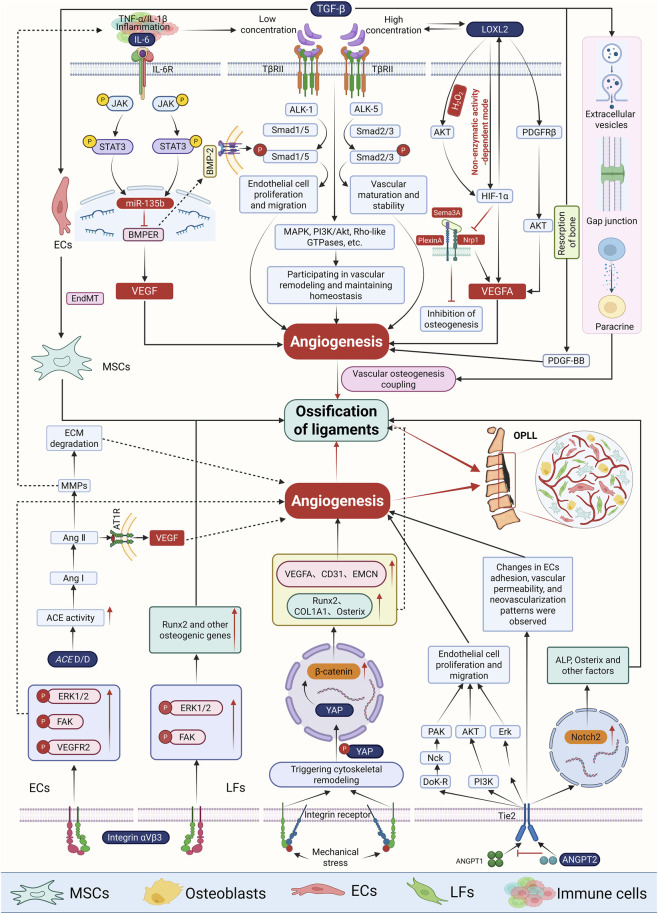
Key molecules and signaling pathways of angiogenesis in OPLL. The development of OPLL is a complex process involving multiple cells and intertwined signaling pathways, where the angiogenesis-osteogenesis coupling serves as the core mechanism driving the transition from fibrosis to ossification. Within the inflammatory/hypoxic microenvironment, LOXL2 enhances ECM stiffness through collagen cross-linking and activates the TGF-β/Smad pathway. Concurrently, it forms a positive feedback loop with HIF-1α and the PDGFRβ-AKT pathway, amplifying VEGFA signaling to promote ECs migration, lumen formation, and ligament ossification. HIF-1α also suppresses Nrp1 expression, thereby inhibiting osteoclastogenesis, promoting osteogenesis, and amplifying angiogenesis signals. Integrin αVβ3, serving as a common hub for vascular and ossification processes, interacts with VEGFR2 to induce ECs migration and tubulogenesis via the FAK/ERK pathway while enhancing Runx2 expression in LFs cells. ANGPT2 regulates angiogenesis by interfering with Tie2 homeostasis and competing with ANGPT1; simultaneously, it upregulates Notch2 to promote expression of osteogenic factors like ALP and Osx, synergistically advancing ossification. Inflammation also significantly promotes endochondral ossification: the IL-6/STAT3 pathway upregulates miR-135b and suppresses BMPER, reshaping the angiogenesis-osteogenesis coupling. TGF-β promotes ligament ossification via EndMT while regulating ECs proliferation and migration through ALK1/ALK5, synergistically promoting angiogenesis with PDGF-BB released during bone resorption. Genetically, *ACE* I/D polymorphism (D/D genotype) enhances angiogenesis and inflammatory responses via the Ang II-AT1R-VEGF pathway and MMPs-mediated matrix remodeling. Mechanical stress, as a trigger for OPLL, synergistically activates angiogenesis and osteogenesis through the YAP/β-catenin pathway, accelerating disease progression. Note: The figure associated with this section was created using BioRender.

OPLL is characterized by endochondral ossification as its predominant pathological mode, with vascular infiltration representing a defining feature of disease progression. RNA sequencing of ligament specimens from patients with OPLL identified 677 differentially expressed genes (DEGs), among which angiogenesis- and osteogenesis-related genes including *SP7*, *ALPL*, and *BGLAP*, were significantly dysregulated. GO and KEGG analyses revealed that these DEGs were enriched in pathways associated with extracellular matrix organization, angiogenesis, and skeletal system development. Consistently, GSEA strongly indicated that angiogenesis is one of the most profoundly altered biological processes in ossified ligament tissue, underscoring its critical role in the initiation and progression of OPLL (Wang et al., 2024). Within this context, the VEGF family serves as a central regulatory axis by inducing endothelial cell proliferation, lumen formation, and increased vascular permeability (Dai and Rabie, 2007). *In vitro* Matrigel assays demonstrated that ligament-derived cells can form capillary-like structures, a process that is significantly enhanced by supplementation with VEGFA or PDGF-BB, but markedly attenuated by inhibition of platelet-derived growth factor receptors (PDGFRs) or vascular endothelial growth factor receptors (VEGFRs). Complementary *in vivo* experiments further showed that subcutaneous implantation of ligament-derived cells mixed with Matrigel into nude mice resulted in the formation of functional vascular networks, providing direct evidence that ligament cells actively participate in angiogenesis and thereby promote OPLL progression (Wang et al., 2024). Collectively, these findings establish angiogenesis as an indispensable component of OPLL pathophysiology. Emerging evidence indicates that multiple signaling pathways converge to regulate this process (Figure 3). Elucidating the precise molecular mechanisms underlying angiogenesis in OPLL will therefore be essential for a comprehensive understanding of disease pathogenesis and for the identification of potential therapeutic targets.

### 4.3 The key molecules and signaling axes of angiogenesis in OPLL

#### 4.3.1 LOXL2/HIF-1α/VEGF signaling axis

The VEGF family comprises VEGF-A, VEGF-B, VEGF-C, VEGF-D, VEGF-E, VEGF-F, placental growth factor (PLGF), and endocrine gland-derived VEGF (EG-VEGF). Their biological effects are mediated primarily through the receptors VEGFR-1, VEGFR-2, VEGFR-3, and the co-receptors neuropilins (NRPs) (Melincovici et al., 2018). Among these, VEGF-A and VEGF-B, together with VEGFR-1, VEGFR-2, and the co-receptors neuropilin-1 (NRP1) and neuropilin-2 (NRP2), constitute the core signaling network governing angiogenesis. VEGF-A is the most critical pro-angiogenic factor in endochondral ossification (Dai and Rabie, 2007), and its expression is tightly regulated by multiple upstream signals, including HIF-1α, BMP-2, and TGF-β (Deckers et al., 2002; Song et al., 2023; Tokuda et al., 2003).

The lysyl oxidase (LOX) family comprises copper-dependent extracellular matrix (ECM) crosslinking enzymes that maintain tissue mechanical stability through catalyzing collagen and elastin crosslinking (Umana-Diaz et al., 2020). Beyond this structural role, LOX family members are increasingly recognized as regulators of angiogenic and osteogenic signaling (Bae et al., 2018; Umana-Diaz et al., 2020; Wang et al., 2024). In angiogenesis, LOX promotes endothelial cell migration and lumen formation by activating the PDGFRβ-AKT pathway and upregulating VEGFA expression (Baker et al., 2013). LOXL2 has been identified as a key hypoxia-induced ECM component in endothelial cells. Sustained HIF-1α signaling upregulates LOXL2 transcription, leading to LOXL2 protein accumulation within the vascular basement membrane, where it co-localizes with type IV collagen and exhibits spatiotemporally restricted expression during both developmental and pathological angiogenesis. Genetic deletion of *LOXL2a* results in impaired blood flow and vascular developmental defects, while *in vitro* knockdown of *LOXL2a* markedly reduces capillary-like structure formation (Bignon et al., 2011). In osteogenic regulation, LOX not only stabilizes the bone matrix through collagen crosslinking but also interacts directly with signaling pathways. LOX can bind specifically to TGF-β1, and LOX overexpression suppresses TGF-β1, induced Smad3 phosphorylation without affecting BMP-2-Smad1/5/8 signaling (Atsawasuwan et al., 2008). Conversely, TGF-β induces LOX expression, and pharmacological inhibition of TGF-β signaling can rescue phenotypes associated with LOX deficiency (Kutchuk et al., 2015), indicating a bidirectional regulatory relationship.

In OPLL tissues, H-type vessels and osteoblasts are markedly increased, accompanied by high LOXL2 expression, positioning LOXL2 as a critical molecular node linking aberrant angiogenesis and osteogenic activation (Wang et al., 2024). Gain- and loss-of-function studies have demonstrated that LOXL2 enhances HIF-1α transcription through mechanisms independent of its enzymatic activity, thereby activating downstream VEGFA and PDGF-BB expression and promoting the transition of ligament cells toward an endothelial-like phenotype. Conversely, LOXL2 inhibition significantly downregulates VEGFR and PDGFR expression, reduces neovascular formation and new bone deposition, and ultimately delays OPLL progression (Wang et al., 2024). Given that OPLL lesions typically reside in a microenvironment characterized by chronic inflammation and hypoxia, sustained stabilization of HIF-1α reinforces VEGFA transcription and reshapes the angiogenic niche, driving ligament-derived cells toward osteogenic and chondrogenic lineages (Dai and Rabie, 2007; Lu et al., 2025; Wang et al., 2024). Within this context, LOXL2 further amplifies signaling through the LOXL2/HIF-1α/VEGFA axis by stabilizing HIF-1α, cooperating with TGF-β signaling, and remodeling the ECM. This axis also integrates with inflammatory angiogenic signals such as IL-6 and IL-1β (Laczko and Csiszar, 2020), forming a self-reinforcing “inflammation-hypoxia-angiogenesis-ossification” feedback loop. Consequently, targeting the LOXL2/HIF-1α/VEGFA signaling axis may represent a promising therapeutic strategy to simultaneously suppress pathological angiogenesis and attenuate heterotopic ossification in OPLL.

#### 4.3.2 Sema3A/Nrp1 signaling axis

Semaphorin-3A (Sema3A) is secreted by osteoblasts and sensory nerve terminals. During physiological bone remodeling, Sema3A exerts bidirectional regulation of bone metabolism by binding to the neuropilin-1 (Nrp1)/PlexinA receptor complex expressed on osteoblasts, osteoclast precursors, and endothelial cells. On the one hand, activation of the Sema3A/Nrp1 axis suppresses osteoclast differentiation by inhibiting the ITAM-PLCγ2-Ca^2+^-NFATc1 co-stimulatory signaling pathway. On the other hand, Sema3A/Nrp1 promotes osteoblast differentiation through activation of the Wnt/β-catenin pathway and upregulation of osteogenic genes, including *Runx2*, *Sp7*, *ALP*, and *OCN*, thereby conferring a net bone-protective effect characterized by reduced bone resorption and enhanced bone formation (Li et al., 2017).

In heterotopic ossification, the Sema3A/Nrp1 pathway is regarded as a key signaling axis governing angiogenesis-osteogenesis coupling (Huang et al., 2020). Under pathological conditions induced by trauma, inflammation, or chronic mechanical stress, HIF-1α upregulates osteogenic and angiogenic mediators such as BMPs and VEGF while concomitantly downregulating Nrp1 expression. This shift both attenuates the inhibitory effect of Sema3A/Nrp1 on osteoclastogenesis and amplifies pathological angiogenesis through enhanced Nrp1/VEGF signaling, ultimately facilitating ectopic bone formation (Huang et al., 2020; Pellet-Many et al., 2011). In parallel, neuron-derived Sema3A can act via Nrp1 to promote osteogenic differentiation and enrichment of type-H vessels, providing a molecular basis for coordinated “neuro-vascular-bone” remodeling within ossified lesions (Han et al., 2023). At the histological level, Sema3A has been shown to induce ligament cells in tissues such as the periodontal ligament to acquire mesenchymal stem cell-like properties and to exhibit enhanced osteogenic differentiation potential (Wada et al., 2014). OPLL, which arises in the context of chronic abnormal mechanical loading, hypoxia, and inflammation, shares substantial pathological similarities with other forms of heterotopic ossification (Huang et al., 2020; Sato et al., 2007). Given that the posterior longitudinal ligament and the periodontal ligament are both ligamentous connective tissues, it is plausible that Sema3A similarly regulates tendon/ligament stem/progenitor cell differentiation in OPLL. In concert with Nrp1/VEGF-mediated type-H angiogenesis, Sema3A may therefore contribute to angiogenesis-osteogenesis coupling during OPLL progression. Although direct experimental evidence for the role of the Sema3A/Nrp1 axis in OPLL remains limited, extensive findings from studies of heterotopic ossification and bone metabolism support its central function in coordinating angiogenesis and osteogenesis. Further investigation of this pathway in OPLL is warranted and may yield new insights into disease pathogenesis and potential targets for therapeutic intervention.

#### 4.3.3 Integrin αVβ3/FAK/ERK signaling axis

Integrin αVβ3 is a cell adhesion receptor formed by the noncovalent association of the αV and β3 transmembrane subunits. Each subunit contains a large extracellular domain, a single-pass transmembrane region, and a short cytoplasmic tail. The extracellular domains bind a wide range of ligands containing the RGD (Arg-Gly-Asp) motif, including bone-related proteins, fibrinogen, angiopoietins, and multiple ECM components (Hynes, 2002). Upon engagement with RGD-containing ligands, integrin αVβ3 transduces extracellular cues into intracellular signals, activating pathways such as FAK and ERK, thereby regulating diverse cellular processes including proliferation, migration, differentiation, and apoptosis (Chastney et al., 2025; Hynes, 2002). Because integrin αVβ3 is widely expressed in osteoblasts and ECs, it plays a critical role not only in bone formation and remodeling but also in angiogenesis and inflammatory responses (Hynes, 2002; Mahabeleshwar et al., 2007). Recent studies have shown that integrin αVβ3 is significantly upregulated in LFs derived from patients with OPLL, where it promotes osteogenic phenotypic conversion. *In vitro* experiments demonstrated that αVβ3 activates the FAK/ERK1/2 signaling cascade, induces expression of the key osteogenic transcription factor Runx2, enhances ALP activity, and promotes mineralized nodule formation. Conversely, blockade of αVβ3 suppresses activation of this pathway, reduces Runx2 expression, and markedly attenuates the osteogenic differentiation capacity of LFs (Geng et al., 2024). In parallel, ligament tissues from patients with OPLL exhibit a pronounced increase in CD31-positive microvessels, indicating active involvement of ECs in shaping the angiogenic-osteogenic microenvironment (Ichikawa et al., 2021; Wang et al., 2024). Integrin αVβ3 is highly expressed on ECs, where its interaction with VEGFR2 enhances endothelial migration and tube formation, accompanied by increased phosphorylation of VEGFR2, FAK, and ERK1/2. Specific inhibition of αVβ3 markedly impairs EC migration and angiogenic capacity and suppresses these phosphorylation events, indicating that αVβ3 is a key regulator of angiogenesis within OPLL lesions (Geng et al., 2024). Animal studies further confirm the dual role of integrin αVβ3 in OPLL pathogenesis. Blockade of αVβ3 not only reduces bone volume fraction (BV/TV) and BMD within ectopic ossification sites but also significantly decreases the numbers of osteogenic marker-positive (OCN^+^) cells and vascular marker-positive (CD31^+^) cells. These findings demonstrate that targeting αVβ3 can simultaneously suppress osteogenesis and angiogenesis *in vivo* (Geng et al., 2024). Collectively, integrin αVβ3 functions as a critical signaling integrator that maintains angiogenesis-osteogenesis coupling in OPLL, offering new mechanistic insight into the molecular basis of disease initiation and progression.

#### 4.3.4 ANGPT/notch signaling axis

The angiopoietin (ANGPT) family comprises several key members, including ANGPT1 and ANGPT2. Among them, ANGPT2 is generally regarded as a vascular “plasticity” factor that competitively binds to its receptor Tie2, thereby destabilizing the ANGPT1/Tie2 axis and modulating EC adhesion, vascular permeability, and patterns of neovessel growth (Eklund et al., 2017). The Notch signaling pathway mediated by Notch receptors (e.g., Notch1, Notch2, and Notch3) and their ligands from the Delta-like and Jagged families plays broad roles in osteogenesis and angiogenesis. Emerging evidence indicates that, in pathological contexts such as ligament ossification, ANGPT2 not only regulates vascular remodeling but also promotes osteogenic differentiation by upregulating Notch2 signaling and enhancing the expression of downstream osteogenic markers, including ALP and Osterix, thereby markedly accelerating bone matrix deposition (Yang et al., 2018). In TOLF cell models, ANGPT2 expression positively correlates with osteogenic potential, whereas pharmacological inhibition or genetic knockdown of ANGPT2 significantly attenuates ossification (Yang et al., 2018). These findings further suggest a synergistic amplification between the ANGPT2/Tie2 and Notch pathways. On the one hand, ANGPT2 enhances the expression of Notch receptors and ligands; on the other hand, Notch activation provides positive feedback to support both osteogenic differentiation and angiogenic expansion (Peng et al., 2020). Accordingly, coordinated activation of the ANGPT/Tie2/Notch axis may represent a critical signaling mechanism driving ligament ossification, characterized by the simultaneous regulation of EC stability, vascular permeability, and osteogenic initiation signals effects that appear particularly pronounced in Notch2-mediated osteogenic promotion. Moreover, upregulation of ANGPT2 is frequently reinforced by local inflammatory and hypoxic microenvironments. Within OPLL lesions, this reinforcement may enhance angiogenic activity and osteogenic potential, thereby establishing an aberrant “vascular-osteogenic” niche that accelerates pathological progression. Taken together, these observations suggest that therapeutic targeting of the ANGPT/Tie2/Notch signaling axis may suppress pathological angiogenesis and, in turn, slow the progression of OPLL.

#### 4.3.5 IL-6/Stat3/miR-135b signaling axis

IL-6 is a key pro-inflammatory cytokine that exerts its biological effects by binding to the IL-6 receptor (IL-6R), thereby activating downstream Janus kinases (JAKs) and inducing phosphorylation and nuclear translocation of signal transducer and activator of transcription-3 (STAT3), which initiates transcription of target genes (Mihara et al., 2012). This signaling axis plays a pivotal role in inflammation-associated angiogenesis and osteogenesis (Ji and Yu, 2023; Lamy et al., 2012) and contributes to aberrant regulation of the vascular microenvironment within OPLL lesions. MicroRNA-135b (miR-135b) is a key downstream effector of the IL-6/STAT3 pathway. Encoded at the LEM domain-containing protein 1 (LEMD1) locus and transcribed from the *miR-135B* gene (1q32.1), miR-135b is widely expressed across multiple tissues and organs (Cao et al., 2020; Kadkhoda et al., 2022; Shao et al., 2024). By binding complementary sequences in target mRNAs, miR-135b suppresses translation or promotes mRNA degradation, thereby regulating biological processes relevant to cardiovascular and cerebrovascular systems. Previous studies have shown that miR-135b promotes angiogenesis by inhibiting expression of phosphoinositide-3-kinase regulatory subunit 2 (PIK3R2) or von Hippel-Lindau protein (VHL) (Liu et al., 2021; Zhang and Zhang, 2022). Bone morphogenetic protein-binding endothelial cell precursor-derived regulator (BMPER) was originally identified through differential protein screening in endothelial precursor cells during *Drosophila* embryogenesis. BMPER binds and modulates at least three bone morphogenetic proteins (BMP-2, BMP-4, and BMP-6) and can exert either stimulatory or antagonistic effects on BMP signaling depending on the biological context (Moser et al., 2003). Loss-of-function studies suggest that BMPER primarily acts as a positive BMP modulator by facilitating the interaction between BMPs and their cognate receptors (Heinke et al., 2008; Serpe et al., 2008). Activation of the BMP/Smad pathway is a critical step in osteogenic differentiation. Upon BMP binding to BMP receptors on the membrane of bone marrow mesenchymal stem cells (BMSCs), Smad1/5/8 are phosphorylated and form a complex with Smad4, which translocates into the nucleus to induce expression of osteogenic transcription factors such as Runx2 and other downstream targets, including *COL1A1*, *OCN*, and *OSX*. *In vitro* studies have demonstrated that BMPER overexpression significantly enhances BMP-2-induced phosphorylation of Smad1/5/8 and upregulates osteogenic markers in human BMSCs, whereas BMPER inhibition effectively suppresses these effects (Xiao et al., 2018). Beyond its role in osteogenesis, BMPER also actively participates in angiogenesis. BMPER upregulates VEGF expression while suppressing endostatin. Conditioned medium from BMPER-overexpressing human BMSCs markedly increases migration, branching, and capillary-like structure formation in human umbilical vein endothelial cells (HUVECs), whereas conditioned medium from BMPER-silenced BMSCs significantly attenuates these angiogenic responses. *In vivo* studies further confirm that BMPER overexpression in heterotopic ossification models enhances both neovascularization and bone formation (Xiao et al., 2018). Collectively, these findings identify BMPER as a positive regulator of angiogenesis-osteogenesis coupling. By potentiating BMP-2-driven osteogenic differentiation while simultaneously promoting angiogenesis, BMPER plays a crucial role in coordinating vascular and skeletal responses during ectopic bone formation.

During the pathogenesis of OPLL, the IL-6/STAT3 signaling pathway has been shown to exert complex and context-dependent regulatory effects. Clinical studies have demonstrated that serum IL-6 levels are significantly elevated in patients with OPLL and are associated with increased expression of *Sox9*, *Runx2*, and *Sirt1*. Immunohistochemical analyses further revealed that these factors are localized to mesenchymal cells within degenerated regions of the ligament matrix and are situated adjacent to the ossification front, suggesting a direct involvement of IL-6 signaling in early endochondral ossification (Saito et al., 2023). In contrast, other studies have reported that IL-6, through a STAT3-dependent mechanism, upregulates miR-135b, which in turn suppresses the expression of BMP-binding endothelial cell precursor-derived regulator (BMPER) (Ji and Yu, 2023). Given that BMPER positively regulates angiogenesis-osteogenesis coupling by enhancing osteogenic and angiogenic gene expression in human bone marrow mesenchymal stem cells (hBMSCs) (Xiao et al., 2018), inhibition of BMPER would be expected to disrupt coordinated angiogenesis and osteogenesis. Indeed, suppression of BMPER has been shown to attenuate cyclic tensile strain-induced OPLL progression (Ji and Yu, 2023), a finding that appears to diverge from the pro-ossification effects reported by Saito (Saito et al., 2023). Similar paradoxical effects of IL-6 have also been observed in murine arthritis models and *in vitro* co-culture systems of chondrocytes and osteoblasts, where excessive IL-6 expression induces exaggerated inflammatory responses, promotes tissue damage and articular cartilage degeneration, and ultimately suppresses ossification or accelerates cartilage breakdown (Latourte et al., 2017; Song et al., 2021; Sugita et al., 1993).

Based on these observations, we propose that the apparent dual pro- and anti-ossification roles of IL-6 may arise from several non-mutually exclusive factors. First, model-specific differences are likely critical: OPLL represents ectopic ossification within ligamentous tissue and, although it proceeds via endochondral ossification, differs fundamentally from degenerative cartilage disorders in both cellular composition and biomechanical context. Consequently, IL-6 may exert divergent effects in ligament ossification versus articular cartilage degeneration. Second, IL-6 signaling may be highly dependent on concentration and temporal dynamics. Low-level or transient IL-6 exposure may promote angiogenesis and expansion of osteogenic progenitors, whereas sustained or excessive IL-6 signaling may disrupt inflammatory homeostasis and compromise cartilage integrity. Third, the biological outcome of IL-6 signaling is strongly influenced by its interaction with other inflammatory mediators, such as TNF-α and IL-1β, as well as by regulatory molecules including soluble gp130 (sgp130), which modulates classical versus trans-signaling pathways (Mihara et al., 2012).

In summary, the involvement of BMPER in angiogenesis-osteogenesis coupling further supports a central role for the IL-6/STAT3/miR-135b signaling axis in regulating the vascular microenvironment of OPLL lesions. However, several critical questions remain unresolved, including whether IL-6 exerts dose-dependent bidirectional effects, whether miR-135b targets additional angiogenic regulators, and whether BMPER function is modulated by the unique inflammatory and hypoxic milieu of OPLL. Addressing these issues will be essential for elucidating the dynamic regulation of inflammation-angiogenesis interactions in OPLL and may provide a stronger theoretical foundation for therapeutic strategies targeting the IL-6/STAT3/miR-135b axis.

#### 4.3.6 TGF-β/ALK/smad signaling axis

Members of the TGF-β superfamily include TGF-β1/2/3, BMPs, activins, and growth differentiation factors (GDFs), all of which share a highly conserved signaling architecture. TGF-β ligands exert their biological effects by binding to type I and type II serine/threonine kinase receptors, thereby activating the canonical Smad-dependent signaling cascade and regulating a wide spectrum of cellular processes (Pardali et al., 2010; Shi and Massagué, 2003). Notably, TGF-β signaling exhibits a dose-dependent, bidirectional regulatory role in angiogenesis. At low concentrations, TGF-β preferentially activates the ALK1-endoglin-Smad1/5/8 pathway, promoting endothelial cell (EC) proliferation, migration, and lumen formation. In contrast, higher levels of TGF-β favor ALK5-Smad2/3 signaling, which enhances vessel maturation and stabilization, stimulates extracellular matrix (ECM) production, and suppresses excessive EC activity (Goumans et al., 2002; Goumans et al., 2003). In addition, TGF-β can signal through noncanonical pathways, including MAPK, PI3K/Akt, and Rho-like GTPases, to regulate vascular wall cell function and contribute to vascular remodeling and homeostasis (Pardali et al., 2010). Collectively, this multilayered regulatory network plays a pivotal role in maintaining skeletal-vascular homeostasis, in which angiogenesis and osteogenesis are tightly coupled, providing not only metabolic support but also vascular-derived signals that directly modulate bone metabolism (Chen et al., 2020; Li et al., 2025).

Aberrant activation of TGF-β signaling is considered a major driver of OPLL pathogenesis. During the early stages of disease, inflammation- and injury-induced cytokine release activates TGF-β signaling, which promotes differentiation of MSCs into chondrocytes and subsequently initiates endochondral ossification (Takács et al., 2013; Zhang et al., 2020). Evidence indicates that TGF-β expression is upregulated during ligament injury repair, and that its active form can be released during bone resorption, further reinforcing local chondrogenesis and angiogenesis and establishing a pathological positive-feedback loop linking vascular invasion and ossification (Wang X. et al., 2018; Yu et al., 2021). Thus, TGF-β may contribute to OPLL progression through multiple, interconnected mechanisms. First, TGF-β binding to ALK1 or ALK5 receptors differentially activates Smad1/5/8 or Smad2/3 signaling, thereby regulating EC proliferation, migration, and pericyte recruitment to drive neovascular formation; excessive activation of Smad2/3 signaling may also enhance osteogenic transcriptional programs (Goumans et al., 2002; Zhang et al., 2025). Second, TGF-β can induce endothelial-to-mesenchymal transition (EndMT), enabling ECs to acquire osteogenic potential, a process proposed as one cellular source contributing to posterior longitudinal ligament ossification (Ma et al., 2020). Third, TGF-β enhances signaling mediated by platelet-derived growth factor-BB (PDGF-BB) released during bone resorption, thereby promoting the formation of type-H vessels. These vessels are markedly enriched in OPLL lesions and exhibit strong coupling with osteogenesis (Li et al., 2025; Wang et al., 2024; Wang X. et al., 2018). Moreover, during the early phases of ossification, injury-induced immune cell infiltration combined with aberrant TGF-β activation generates a highly inflammatory and hypervascular microenvironment, which accelerates disease progression (Li et al., 2025; Zhang et al., 2020). Finally, TGF-β signaling can mediate intercellular communication between osteogenic cells and ECs through paracrine signaling, extracellular vesicle transfer, and gap junctions, further reinforcing angiogenesis-osteogenesis coupling (Li et al., 2025).

#### 4.3.7 *ACE* D/D-Ang II-VEGF signaling axis

Angiotensin I-converting enzyme (ACE) is a central component of the renin-angiotensin system (RAS) that catalyzes the conversion of biologically inactive angiotensin I (Ang I) into the potent effector angiotensin II (Ang II), thereby playing a critical role in the regulation of vascular tone, inflammatory responses, electrolyte homeostasis, and tissue remodeling (Soubrier, 2013). The *ACE* gene is located on chromosome 17q23 and comprises 26 exons and 25 introns. A well-characterized insertion/deletion (I/D) polymorphism (rs4646994), defined by the presence or absence of a 287-bp Alu repeat sequence in intron 16, is closely associated with ACE activity. Individuals carrying the D allele particularly those with the D/D genotype exhibit significantly higher plasma ACE levels than carriers of other genotypes (Rigat et al., 1990). The *ACE* I/D polymorphism has been linked to susceptibility to multiple cardiovascular and renal disorders (Amara et al., 2018; Soubrier, 2013). Genetic association studies have further demonstrated that the frequency of the *ACE* D/D genotype is significantly higher in patients with OPLL than in healthy controls, suggesting that this genotype may confer genetic susceptibility to OPLL (Kim et al., 2014). Mechanistically, Ang II generated by ACE activates the angiotensin II type 1 receptor (AT1R), leading to upregulation of VEGF expression and subsequent stimulation of endothelial cell proliferation, migration, and neovascular formation (Carbajo-Lozoya et al., 2012). In addition, elevated Ang II levels enhance the expression of MMPs, which degrade the ECM and create permissive migratory pathways for invading endothelial cells (Tamarat et al., 2002). During ECM degradation, MMPs can further stimulate the production and release of inflammatory cytokines such as IL-6 and TNF-α and increase VEGF bioavailability, thereby reinforcing a pro-angiogenic microenvironment (Bergers et al., 2000; Manicone and McGuire, 2008; Rundhaug, 2005). Given the central role of angiogenesis-osteogenesis coupling in OPLL pathogenesis, the *ACE* D/D genotype may promote disease progression by enhancing Ang II/VEGF-mediated angiogenesis, which in turn facilitates ectopic ossification. However, the precise molecular and cellular mechanisms linking ACE polymorphism, angiogenic activation, and ligament ossification remain to be elucidated and warrant further investigation.

#### 4.3.8 YAP/β-catenin signaling axis

During angiogenesis and tendon/ligament ossification, the Yes-associated protein (YAP)/β-catenin signaling axis functions as a central regulatory hub. Activation of this axis has been shown to promote EC proliferation and migration by upregulating angiogenic markers such as VEGFA, CD31, and EMCN, thereby driving the formation of type-H vessels. Concurrently, during osteogenic differentiation of TDSCs, YAP/β-catenin signaling enhances mineralization and accelerates osteogenesis through increased expression of osteogenic genes, including *ALP*, *OPN*, *OCN*, *Runx2*, and *BSP* (Luo et al., 2023). Pharmacological or genetic inhibition of YAP reduces β-catenin expression and phosphorylation, markedly impairing angiogenic capacity in HUVECs and osteogenic differentiation in TDSCs. In animal models of heterotopic ossification, YAP inhibition effectively decreases type-H vessel formation and ectopic bone deposition, underscoring the essential role of this signaling axis in angiogenesis-osteogenesis coupling (Luo et al., 2023). Given that OPLL progression similarly depends on coordinated angiogenesis and osteogenesis, the YAP/β-catenin axis is likely to represent a key pathological mechanism. Notably, mechanical stress, an established etiological factor in OPLL can activate YAP signaling through integrin-mediated mechanotransduction. Mechanical stimulation induces cytoskeletal remodeling, leading to YAP dephosphorylation and nuclear translocation, which in turn facilitates β-catenin activation and upregulates osteogenic genes such as *Runx2*, *COL1A1*, *Osterix*, *OCN*, and *ALP* (Stanley et al., 2019; Totaro et al., 2018). This YAP-Wnt/β-catenin-mediated response is particularly pronounced in primary LFs derived from patients with OPLL. Importantly, shRNA-mediated knockdown of YAP or β-catenin significantly attenuates mechanical stretch induced upregulation of osteogenic genes, providing direct evidence that mechanical stress promotes LF osteogenic differentiation through YAP-dependent activation of β-catenin signaling (Zhu et al., 2023).

Collectively, these findings indicate that the YAP/β-catenin signaling axis serves as a pivotal integrator of mechanical stress sensing and angiogenesis-osteogenesis coupling. Aberrant activation of this pathway may represent a key mechanism underlying the amplification of osteogenic and angiogenic signaling within OPLL lesions. By linking external mechanical cues to intracellular transcriptional programs and coordinating functional crosstalk between ECs and osteogenic cells, the YAP/β-catenin axis emerges as a highly promising target for further investigation into the pathogenesis and potential treatment of OPLL.

### 4.4 Mechanism integration

The pathological core of OPLL is characterized by a cascading and self-amplifying process involving inflammation/hypoxia-angiogenesis-endochondral ossification, which is achieved through the coordinated integration of multiple functional signaling pathways.

#### 4.4.1 Hypoxia and the HIF-1α/VEGFA axis

A hypoxic microenvironment stabilizes HIF-1α, which cooperates with LOXL2 and the ACE D/D-Ang II axis to upregulate angiogenic and osteogenic mediators such as VEGFA and PDGF-BB. This signaling cascade promotes EC migration, neovessel formation, and enrichment of type-H vessels, while concurrently directing ligament-derived cells toward osteogenic and chondrogenic lineages (Carbajo-Lozoya et al., 2012; Kim et al., 2014; Lu et al., 2025; Wang et al., 2024).

#### 4.4.2 Endothelial-osteogenic communication networks

Angiogenesis-osteogenesis coupling is orchestrated by a group of interrelated pathways that together constitute a functional “vascular-ossification unit,” including ANGPT2/Tie2/Notch2, integrin αVβ3/FAK/ERK, Sema3A/Nrp1, and YAP/β-catenin signaling. ANGPT2 competitively disrupts ANGPT1-Tie2 signaling, induces Notch2 activation, enhances expression of osteogenic markers such as ALP and Osterix, and modulates EC stability and vascular permeability (Peng et al., 2020; Yang et al., 2018). Integrin αVβ3 activates the FAK/ERK pathway to induce Runx2 and ALP expression and mineralized nodule formation in ligament fibroblasts, while synergizing with VEGFR2 to enhance EC migration and angiogenesis (Geng et al., 2024). Sema3A/Nrp1 signaling suppresses osteoclast differentiation and activates Wnt/β-catenin signaling, promotes osteogenic gene expression, and induces ligament cells to acquire mesenchymal stem cell-like properties (Li et al., 2017; Wada et al., 2014). Meanwhile, YAP/β-catenin acts as a central mechanosensitive hub regulating both type-H vessel formation and osteogenic differentiation of tendon-derived stem cells and ligament fibroblasts (Luo et al., 2023).

#### 4.4.3 Inflammatory regulation via IL-6/STAT3 signaling

Inflammatory stimulation activates the IL-6/STAT3 pathway, which modulates angiogenesis-osteogenesis coupling by upregulating miR-135b and suppressing BMPER expression. Through dynamic regulation of BMP/Smad and VEGF signaling, this axis exhibits context-dependent, bidirectional effects, either promoting or restraining ossification, depending on disease model, inflammatory intensity, and temporal dynamics (Ji and Yu, 2023; Latourte et al., 2017; Saito et al., 2023; Song et al., 2021; Xiao et al., 2018).

#### 4.4.4 Growth factor signaling and vascular remodeling

The TGF-β/Smad pathway regulates chondrogenesis, eEndMT, and type-H vessel formation through signaling switches between ALK1 and ALK5 (Goumans et al., 2002; Ma et al., 2020; Wang X. et al., 2018; Li et al., 2025). BMPER, acting as a positive modulator of BMP-2 signaling, enhances VEGF expression, EC migration, and capillary-like structure formation, while simultaneously reinforcing osteogenic differentiation and matrix mineralization (Xiao et al., 2018).

Collectively, OPLL progression depends on a multilayered, feedback-reinforced angiogenesis-osteogenesis coupling network that integrates hypoxia, inflammation, extracellular matrix remodeling, and mechanical stress. Systematic elucidation of this integrated signaling architecture provides a robust theoretical framework for understanding OPLL pathogenesis and for developing targeted therapeutic strategies aimed at disrupting pathological vascular-osteogenic coupling.

## 5 Current treatment methods and future targeted strategies for OPLL

### 5.1 Existing treatment methods

OPLL most commonly affects the cervical spine and frequently results in spinal cord compression and neurological dysfunction. In severe cases, even minor trauma may precipitate catastrophic neurological injury, rendering clinical management particularly challenging. For patients who are asymptomatic or only mildly symptomatic, conservative treatment may be considered as an initial approach (Bellaire et al., 2025); however, close surveillance is essential because disease progression is not uncommon. Longitudinal data indicate that approximately 16.7% of patients without myelopathy at presentation develop myelopathy during conservative management (Pham et al., 2011). Accordingly, patients undergoing nonoperative treatment require careful follow-up and dynamic neurological assessment, with timely conversion to surgical decompression once progressive radiculopathy or spinal cord compression is identified. Surgical strategies for cervical OPLL include anterior, posterior, and combined approaches (Sun et al., 2024). Anterior procedures offer the advantages of direct decompression, relatively limited surgical trauma, and the potential to maintain or restore cervical lordosis (Park et al., 2023). Common anterior techniques include anterior cervical corpectomy and fusion (ACCF), anterior cervical discectomy and fusion (ACDF), and vertebral body sliding osteotomy (VBSO). However, anterior surgery is also associated with procedure-specific risks, including esophageal injury, dysphagia, hoarseness, dural tears, graft subsidence, epidural hematoma, and pseudarthrosis (Kim et al., 2019). In recent years, technical advances such as anterior controllable antedisplacement and fusion (ACAF), anterior cervical ossified posterior longitudinal ligament *en bloc* resection (ACOE) (Sun et al., 2024), and minimally invasive endoscopic techniques have shown promise in improving decompression efficacy while reducing the risk of spinal cord injury and postoperative complications, thereby expanding options for individualized and precision-based treatment. Posterior approaches, including laminectomy and laminoplasty, achieve indirect decompression and are generally associated with greater procedural safety and lower complication rates compared with anterior surgery (Zhou et al., 2025). Nevertheless, residual ossified lesions may continue to progress following posterior decompression, occasionally necessitating secondary anterior surgery. In addition, postoperative complications such as cervical kyphotic deformity remain concerns (He et al., 2024; Sun et al., 2024). Combined anterior-posterior approaches are reserved for complex cases in which adequate decompression cannot be achieved through a single corridor. While this strategy integrates the benefits of both direct and indirect decompression and facilitates reconstruction of spinal stability, it is limited by increased surgical trauma and higher costs, underscoring the need for strict patient selection (He et al., 2024). At present, the optimal surgical strategy for cervical OPLL remains controversial, and clinical decision-making must be individualized based on patient comorbidities, ossification morphology, and disease severity. Importantly, surgical exposure is often limited, complete removal of ossified lesions may be difficult, and postoperative recurrence remains a concern. Moreover, effective conservative therapies are scarce. These limitations highlight the urgent need to develop mechanism-based, targeted therapeutic strategies that may complement surgical intervention, improve clinical outcomes, or delay disease progression.

### 5.2 Exploration of novel treatments for OPLL based on potential therapies for ectopic ossification

OPLL is generally regarded as a distinct subtype of heterotopic ossification, and the two conditions share several common pathogenic mechanisms. Beyond conventional anti-inflammatory management, a variety of emerging non-surgical therapeutic strategies have been proposed in recent years, including: (1) low-dose radiotherapy; (2) inhibitors of bone morphogenetic protein (BMP) signaling; (3) retinoic acid receptor-γ (RARγ) agonists; (4) Wnt signaling inhibitors; (5) hypoxia-inducible factor-1α (HIF-1α) pathway inhibitors; (6) combined therapy using tissue-nonspecific alkaline phosphatase (TNAP) inhibitors and pyrophosphate (PPi); (7) small extracellular vesicle (sEV)-microRNA (miRNA)-based interventions; (8) miRNA-specific targeted regulation; (9) histamine H2 receptor antagonists (H2RAs). Although most of these approaches remain at the preclinical or early clinical investigation stage, several have demonstrated promising efficacy in both *in vitro* and *in vivo* models. However, their applicability, safety, and therapeutic value in OPLL specifically have yet to be rigorously validated. Collectively, these multi-target and multi-mechanistic strategies not only offer potential new avenues for non-surgical management of OPLL but also broaden the therapeutic landscape for ossification-related disorders more generally (Table 1).

**TABLE 1:** Novel intervention strategies for OPLL based on potential therapies for ectopic ossification.

Measures	Represents drugs or small molecules	Mechanism	Explanation	Strengths and weaknesses	References
Low-dose radiotherapy	—	By down-regulating the BMP and ALK4 signaling pathways, the differentiation of bone progenitor cells into fibroblasts is inhibited, thereby reducing abnormal endochondral ossification.	It is mainly used to prevent recurrence after HO surgery. At present, no research has confirmed its therapeutic effect on OPLL. In the future, its adaptability and safety need to be further verified.	Although its toxicity is relatively low and side effects are not common, in recent years, concerns about radiotherapy-induced carcinogenesis and secondary tumors have gradually increased. Especially when used in young patients, the risk/benefit ratio needs to be strictly evaluated.	Georhakopoulos et al. (2020), Hsieh et al. (2025), Wong et al. (2020)
BMP signaling pathway inhibitor	Dorsomorphin and its derivatives (CDN-193189, CDN-212854)	By inhibiting ALK2/3 mediated Smad phosphorylation, the extent of ossification and functional disorders are reduced.	1. The BMP pathway also plays a key regulatory role in OPLL. 2. Multiple inhibition strategies targeting the BMP/Smad axis, especially ALK2/3, may offer promising non-surgical treatment options for OPLL.	It has certain toxicity and side effects, and its clinical transformation is limited.	Tanaka et al. (2001), Williams and Bullock (2018), Yonemori et al. (1997), Yu et al. (2008)
RARγ agonist	Palovarotene	It inhibits osteogenic differentiation, angiogenesis and inflammatory response through pathways such as PI3K-Akt, PPAR, P53, VEGF, Smad and NF-κB, thereby slowing down the process of HO.	OPLL and HO share certain commonalities in osteogenic mechanisms, and the application potential of Palovarotene in OPLL should be further developed.	It has shown potential value in HO studies, especially in significantly improving FOP, but there is also a risk of premature closure of the growth plate	Hsiao and Pacifici (2025) , Huang et al. (2022a) , Huang et al. (2022b)
Wnt pathway inhibitor	Dkk1	By blocking the Wnt/β-catenin signaling pathway, osteogenic transformation of ligament cells is inhibited.	1. The abnormal activation of Wnt/β-catenin signaling is involved in the pathological process of OPLL. 2. The expression of the endogenous inhibitory factor Dkk1 of Wnt is decreased in the serum of patients with OPLL.	It may affect bone metabolism throughout the body	Dong et al. (2020), Niu et al. (2017), Shi et al. (2016)
HIF-1α pathway inhibitor	PX-478、imatinib、Apigenin、Rapamycin	Effectively inhibit the activity of HIF-1α and its downstream osteogenic and angiogenic signaling pathways, thereby slowing down HO.	After activation, HIF-1α promotes angiogenesis and bone formation by regulating genes such as BMP, VEGF and NRP-1, and accelerates HO.	It has a remarkable effect in the FOP model and may be transferred to the OPLL study for application.	Huang et al. (2020), Qureshi et al. (2017), Wang et al. (2016)
TNAP inhibitor + PPi	Levamisole + Exogenous PPi	Targeting pyrophosphate metabolism, it significantly inhibits the pathological process of the OPLL model.	PPi is a natural mineralization inhibitor in the body, but its exogenous form is easily hydrolyzed by TNAP.	There are no side effects such as osteoporosis.	Hiratsuka et al. (2018)
sEVs-miRNAs	miR-320e	miR-320e inhibits TAK1, disrupts the local bone metabolic balance and promotes ectopic bone formation.	1. Ligament cells in OPLL patients can secrete sEVs rich in miR-320e. 2. Injection of sEVs derived from OPLL can accelerate the formation of spinal canal osteophytes. Blocking miR-320e or inhibiting the secretion of sEV can significantly alleviate the lesion.	The miR-320e/TAK1 axis is a new target for the treatment of OPLL.	Xu et al. (2022)
Specific targeted regulation of miRNAs	miR-218	Target and inhibit the expression of osteogenic transcription factors Runx2 and COL1A1, and negatively regulate the OPLL process.	The expression level of miR-218 in ligament cells of OPLL patients was significantly lower than that of non-OPLL patients.	—	Gay et al. (2014), Liu et al. (2018), Xu et al. (2016)
	miR-140-5p	Target IGF1R, inhibit its downstream IRS1/PI3K/Akt/mTOR signaling pathway, and suppress the osteogenic differentiation of hMSCs.	miR-140-5p is downregulated in exosomes of OPLL-derived cells.	—	Tang et al. (2022)
H2RAs	famotidine	The expressions of Runx2, OCN, etc., were regulated through the HRH2-cAMP/PKA/CREB pathway to reduce the ALP activity and the formation of mineralized nodules in OPL-Mscs.	HRH2 expression is upregulated in MSCs derived from OPLL patients and participates in the regulatory process of osteogenic differentiation of cells.	It has wide clinical applications. It has the potential to inhibit the progression of OPLL and provides a realistic and feasible direction for drug redevelopment.	Kim et al. (2013), Liu et al. (2017), Wellner-Kienitz et al. (2003), Zhang et al. (2015)

Dkk1, dickkopf-related protein 1; FOP, fibrodysplasia ossificans progressiva; ALK, anaplastic lymphoma kinase; TAK1, transforming growth factor-activated kinase 1; IRSs, insulin receptor substrate; IGF1R, insulin-like growth factor 1 receptor; hMSCs, human mesenchymal stem cells; HRH2, histamine receptor H2; cAMP, cyclic AMP; PKA, protein kinase A; CREB, cAMP-response element binding protein; OPLL-MSCs, MSCs from OPLL patients.

### 5.3 Targeted angiogenesis: exploring new approaches to OPLL treatment

With advancing insights into the pathophysiology of OPLL, angiogenesis-osteogenesis coupling has emerged as a central driver of disease progression. Accordingly, targeting angiogenesis represents a promising therapeutic strategy, and preliminary studies have already provided proof-of-concept evidence supporting its feasibility. Importantly, angiogenic and osteogenic signaling pathways are extensively interconnected through synergistic activation and positive feedback loops. As a result, interventions aimed at a single molecular target are unlikely to effectively disrupt the complex pathological network underlying OPLL. Future therapeutic development should therefore prioritize multi-target, multi-level modulation of tightly coupled inflammation-angiogenesis-ossification pathways. Such strategies may achieve greater efficacy by simultaneously attenuating inflammatory signaling, pathological neovascularization, and aberrant osteogenic activation. In parallel, the application of advanced drug delivery technologies including nanoparticle-based systems, long-acting cyclic peptides, and targeted antibody therapies may substantially enhance drug accumulation and bioavailability within lesion sites. Together, these approaches hold promise for overcoming current limitations in pharmacological treatment and disease-modifying intervention for OPLL. Representative therapeutic agents and their corresponding molecular targets are summarized in Table 2.

**TABLE 2:** OPLL intervention strategies targeting angiogenesis.

Measures	Target	Mechanism	References
Sorafenib	The LOXL2/HIF-1α/VEGF signaling axis	1. Inhibit LoxL2-mediated endothelial-like differentiation of ligament cells and reduce the formation of capillary-like structures; 2. Delay BMP-induced ectopic bone formation (BIO model) and ENPP1-deficient deficient OPLL-like lesions (LSO model); 3. Block VEGFR and PDGFR, disrupt the coupling of angiogenesis and ossification, inhibit the osteogenic differentiation of ligament cells, and reduce H-type angiogenesis.	Bae et al. (2018), Baker et al. (2013), Coral et al. (2013), Wang et al. (2024)
Cyclo (RGDyK)	Integrin αVβ3/FAK/ERK signaling axis	1. Block αVβ3, downregulate the phosphorylation levels of FAK and ERK1/2, inhibit the expression of Runx2, and weaken the osteogenic differentiation of LFs; 2. Reduce the levels of p-VEGFR2, p-FAK and p-ERK1/2, and inhibit the migration of ECs and angiogenesis.	Geng et al. (2024)
ANGPT inhibition or ANGPT knockdown	ANGPT/Notch signal axis	1. By interfering with the stabilizing effect of the ANGPT/Tie2 axis, the adhesion of ECs, vascular permeability and the growth pattern of new blood vessels are altered. 2. By inhibiting the expression of downstream bone formation markers through Tie2/Notch2, ossification can be suppressed.	Yang et al. (2018)
Stat3 activator or overexpression of miR-135b	IL-6/Stat3/miR-135b signal axis	Inhibit the expression of BMPER, disrupt the vascular-ossification coupling mechanism, and suppress osteogenic differentiation.	Ji and Yu (2023), Xiao et al., 2018)
TGF-β inhibition	TGF-β/ALK/Smad signal axis	1. Interfere with the migration, proliferation and pericytes recruitment of ECs, and inhibit the formation of new blood vessels; And indirectly inhibit the activation of Smad2/3, and suppress the ossification process; 2. Interfere with EndMT; 3. Slow down the PDGF-BB signal released after bone resorption, inhibit H-type angiogenesis, and slow down the osteogenic process of OPLL; 4. Inhibit the inflammatory microenvironment; 5. Interfere with the communication of cells related to angiogenesis and thereby affect the remodeling of the bone vascular system.	Goumans et al. (2002), Li et al. (2025), Ma et al. (2020), Wang X. et al. (2018), Zhang et al. (2025), Zhang et al. (2020)
*ACE* targeted therapy	*ACE* D/D-Ang II-VEGF signal axis	Inhibit angiogenesis and the OPLL process.	Brasier (2010), Carbajo-Lozoya et al. (2012), Kim et al. (2014), Rigat et al. (1990), Tamarat et al. (2002)
Verteporfin	YAP/β-catenin signal axis	Inhibiting the expression of osteogenesis-related genes (*Runx2*, *COL1A1*, *OCN*) and angiogenic factors (VEGFA, CD31, ANGPT1) can effectively slow down the heterotopic ossification of tendons or ligaments, and may also inhibit OPLL.	Luo et al. (2023)

Cyclo (RGDyK), Cyclic Arg-Gly-Asp-D-Tyr-Lys.

## 6 Unsolved problems at present

Despite recent advances in understanding angiogenesis-osteogenesis coupling in OPLL, several critical issues remain unresolved: (1) Causality of angiogenesis in ossification initiation.:It remains unclear whether angiogenesis acts as an upstream driver that initiates ossification or represents a secondary, concomitant event during endochondral ossification. Longitudinal and causality-based evidence is still lacking. (2) Heterogeneity among radiological subtypes: The extent to which angiogenic responses differ among distinct radiographic subtypes of OPLL, and how these differences correlate with ossification progression, has not been systematically evaluated. (3) Key cellular lineages and interaction networks: The principal cell populations involved in pro-angiogenic and pro-osteogenic processes and the interaction networks among them, have yet to be directly demonstrated in human OPLL specimens. (4) Role of type-H vessels and the “vascular-ossification unit: The spatial distribution, functional state, and quantitative relationship between type-H vessels, vascular-ossification units, and osteogenic activity in OPLL remain poorly defined, and no standardized assessment framework currently exists. (5) Dynamic regulation of inflammation-angiogenesis-osteogenesis coupling: The temporal and context-dependent regulatory mechanisms linking inflammation, angiogenesis, and osteogenesis, particularly involving pathways such as IL-6 and TGF-β are incompletely understood, and experimentally testable models explaining their dual pro- and anti-ossification effects are lacking. (6) Mechanobiology and extracellular matrix remodeling: Direct evidence from human tissues demonstrating the coupling among mechanical stimuli, extracellular matrix remodeling, and angiogenesis is still insufficient. (7) Clinical translation of angiogenesis-targeted therapies: Currently available angiogenesis-targeted interventions lack validated, safe, and effective clinical translation strategies. Moreover, integration of such approaches with existing treatment and prevention modalities remains inadequate and warrants further investigation.

## 7 Conclusion and future research directions

OPLL is a complex heterotopic ossification disorder whose initiation and progression result from the coordinated interplay of mechanical loading, inflammatory responses, angiogenesis, and stem/progenitor cell differentiation. Beyond providing metabolic support to lesion sites, angiogenesis plays an active and indispensable role in both the onset and progression of ossification. In particular, the involvement of key molecular regulators including LOXL2, Sema3A, integrin αVβ3, ANGPT2, IL-6, TGF-β, the ACE D/D polymorphism, and YAP collectively establishes a pathological microenvironment characterized by aberrant angiogenesis-osteogenesis coupling. These insights position angiogenesis as a promising target for non-surgical intervention in OPLL. However, extensive crosstalk and feedback amplification among these signaling pathways not only increase the complexity of targeted therapy but also create opportunities for rational, multi-pathway combinatorial interventions.

Future research should prioritize several key directions. First, spatial transcriptomic and multi-omics analyses of human OPLL specimens are needed to resolve cellular composition and intercellular interactions *in situ*, validate the existence and organization of the proposed “vascular-ossification unit,” and identify spatially restricted, therapeutically actionable targets. Second, anti-angiogenic and combination treatment strategies tailored to distinct radiological and molecular subtypes of OPLL should be developed, with the goal of establishing a systematic correspondence among imaging phenotypes, molecular signatures, and therapeutic responses to enable precision medicine approaches. Third, prospective studies integrating imaging features, molecular biomarkers, and histopathological characteristics with ossification growth rates, vascular phenotypes, neurological outcomes, and safety profiles are required to rigorously evaluate the risk-benefit balance of combined anti-inflammatory, anti-angiogenic, and anti-osteogenic interventions. Finally, experimental models that more faithfully recapitulate the chronic disease course and heterogeneity of OPLL should be established. Such models should incorporate long-term mechanical loading and extracellular matrix remodeling and be cross validated with human spatial omics data to enhance the translational relevance of mechanistic studies and drug screening efforts.

Addressing these challenges will facilitate the transition of OPLL research toward a framework that is stratifiable, predictable, and therapeutically actionable, thereby providing a stronger scientific foundation for disease modification, progression delay, and the development of effective non-surgical treatment strategies.
