# Supplementary material for: Angiogenesis in ossification of the posterior longitudinal ligament: progress from mechanism to targeted intervention
Source: Front Cell Dev Biol. 2026 Mar 4;14:1707176. doi: 10.3389/fcell.2026.1707176 (PMC12996184; doi:10.3389/fcell.2026.1707176)
Supplement: Supplementary file 1 [file DataSheet1.docx]

Supplementary Material

# Supplementary Data

No data was used for the research described in the article.

# Supplementary Figures and Tables

## Supplementary Figures


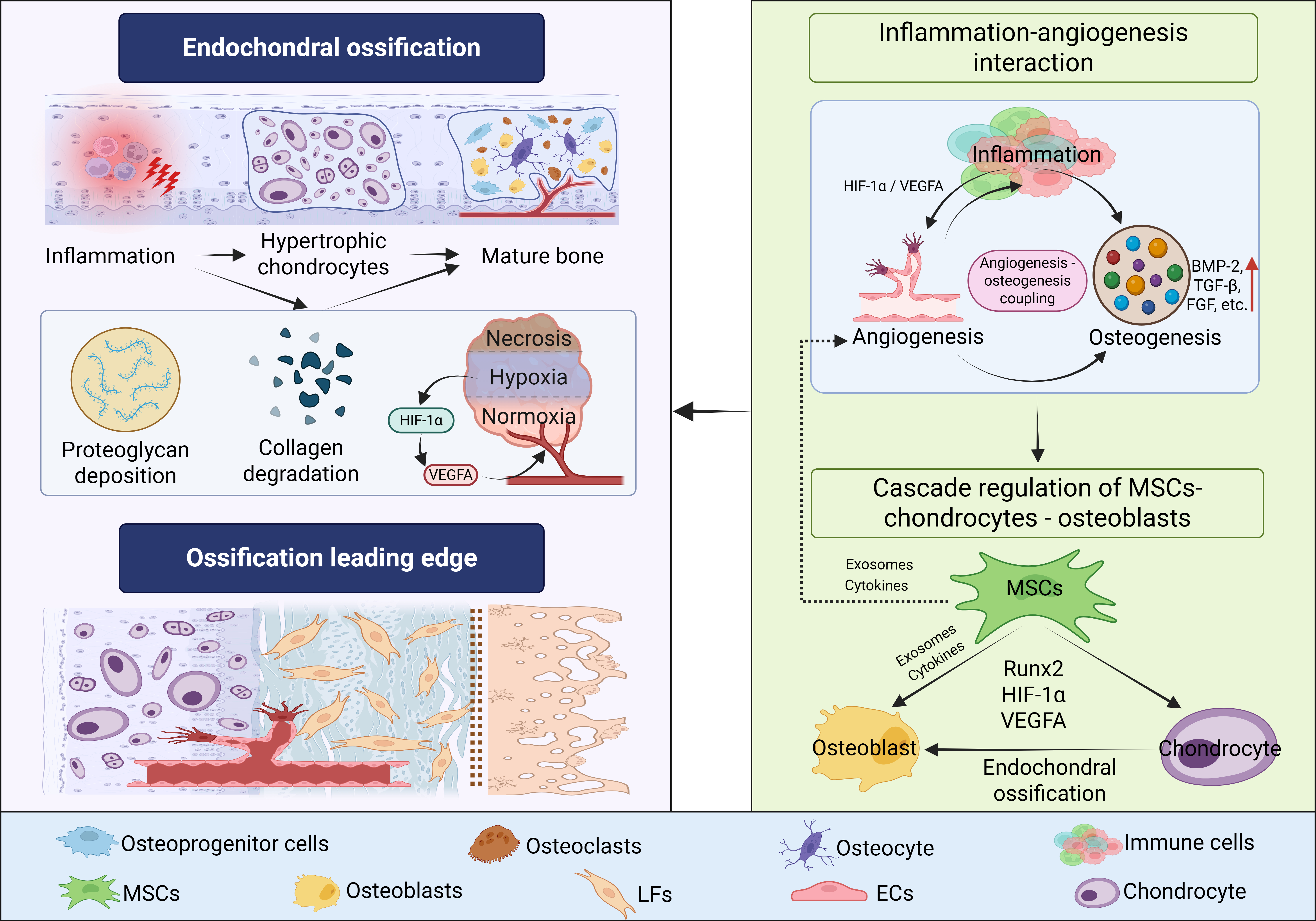


**Supplementary Figure 1. The pathological and cytological characteristics of OPLL.** The pathological changes of OPLL often occur secondary to inflammation. Inflammation stimulation mediates chondrocyte differentiation, proteoglycan deposition, collagen matrix degradation, and angiogenesis, forming a transitional zone where chondroid tissue and fibrotic tissue coexist. Eventually, it transforms into an osteogenic phenotype, promoting ectopic bone formation in ligament tissue. Among them, the cascade regulation of MSCs- chondrocytes - osteoblasts and the "inflammation-angiogenesis interaction" are important factors promoting the progression of OPLL.


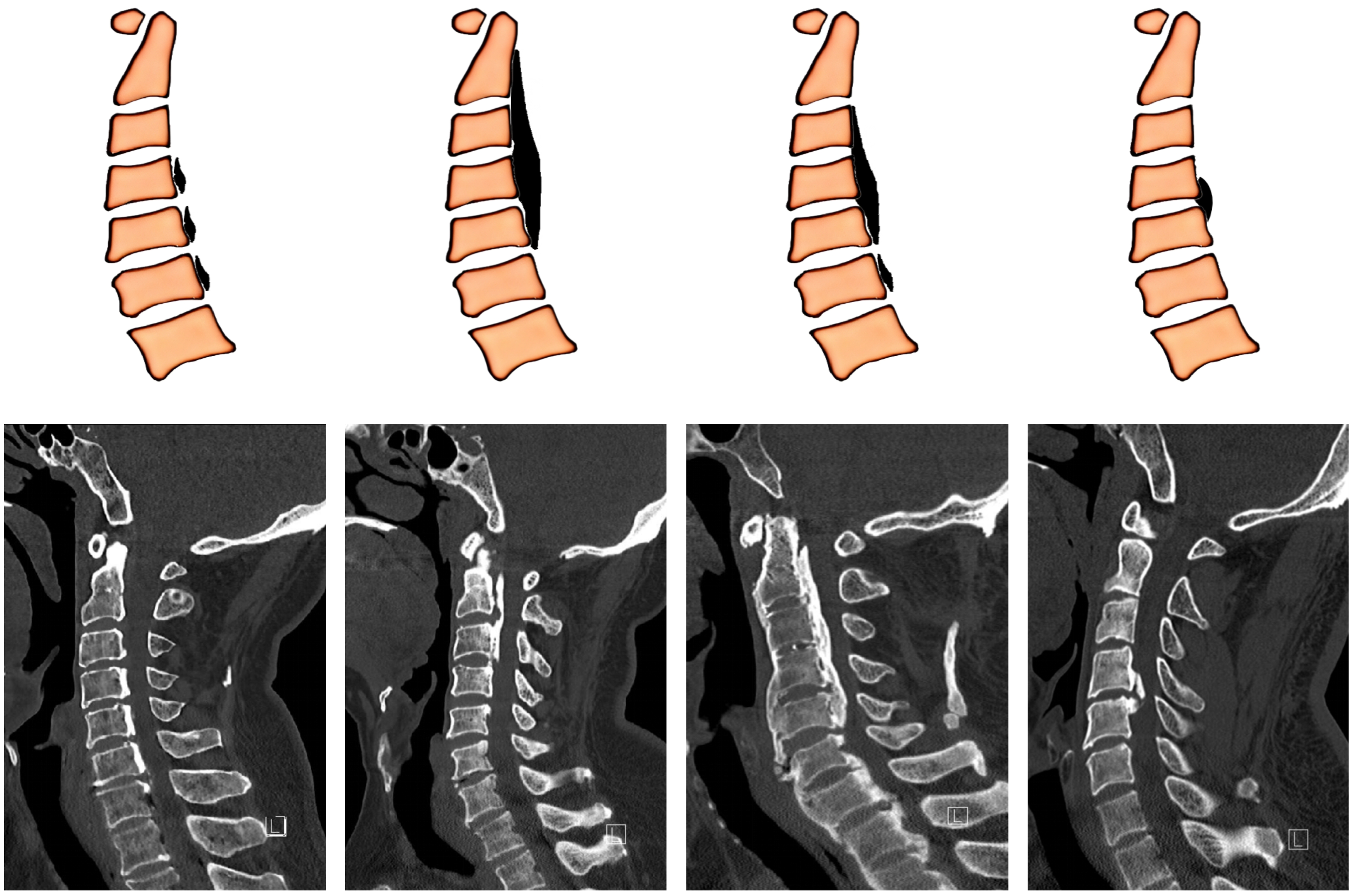


| Segmental type | Continuous type | Mixed type | Localized type |
| --- | --- | --- | --- |

**Supplementary Figure 2. Imaging Classification of OPLL.** Sagittal CT scan images provided by Dr. Lai Jinquan, Shenzhen Luohu Hospital of Traditional Chinese Medicine.





**Supplementary Figure 3. Key molecules and signaling pathways of angiogenesis in OPLL.** The development of OPLL is a complex process involving multiple cells and intertwined signaling pathways, where the angiogenesis-osteogenesis coupling serves as the core mechanism driving the transition from fibrosis to ossification. Within the inflammatory/hypoxic microenvironment, LOXL2 enhances ECM stiffness through collagen cross-linking and activates the TGF-β/Smad pathway. Concurrently, it forms a positive feedback loop with HIF-1α and the PDGFRβ-AKT pathway, amplifying VEGFA signaling to promote ECs migration, lumen formation, and ligament ossification. HIF-1α also suppresses Nrp1 expression, thereby inhibiting osteoclastogenesis, promoting osteogenesis, and amplifying angiogenesis signals. Integrin αVβ3, serving as a common hub for vascular and ossification processes, interacts with VEGFR2 to induce ECs migration and tubulogenesis via the FAK/ERK pathway while enhancing Runx2 expression in LFs cells. ANGPT2 regulates angiogenesis by interfering with Tie2 homeostasis and competing with ANGPT1; simultaneously, it upregulates Notch2 to promote expression of osteogenic factors like ALP and Osx, synergistically advancing ossification. Inflammation also significantly promotes endochondral ossification: the IL-6/STAT3 pathway upregulates miR-135b and suppresses BMPER, reshaping the angiogenesis-osteogenesis coupling. TGF-β promotes ligament ossification via EndMT while regulating ECs proliferation and migration through ALK1/ALK5, synergistically promoting angiogenesis with PDGF-BB released during bone resorption. Genetically, *ACE* I/D polymorphism (D/D genotype) enhances angiogenesis and inflammatory responses via the Ang II-AT1R-VEGF pathway and MMPs-mediated matrix remodeling. Mechanical stress, as a trigger for OPLL, synergistically activates angiogenesis and osteogenesis through the YAP/β-catenin pathway, accelerating disease progression.

## Supplementary Tables

**Supplementary Table 1. Novel intervention strategies for OPLL based on potential therapies for ectopic ossification**

| **Measures** | **Represents drugs or small molecules** | **Mechanism** | **Explanation** | **Strengths and Weaknesses** | **References** |
| --- | --- | --- | --- | --- | --- |
| Low-dose radiotherapy | — | By down-regulating the BMP and ALK4 signaling pathways, the differentiation of bone progenitor cells into fibroblasts is inhibited, thereby reducing abnormal endochondral ossification. | It is mainly used to prevent recurrence after HO surgery. At present, no research has confirmed its therapeutic effect on OPLL. In the future, its adaptability and safety need to be further verified. | Although its toxicity is relatively low and side effects are not common, in recent years, concerns about radiotherapy-induced carcinogenesis and secondary tumors have gradually increased. Especially when used in young patients, the risk/benefit ratio needs to be strictly evaluated. | (Georhakopoulos et al., 2020; Hsieh et al., 2025; Wong et al., 2020) |
| BMP signaling pathway inhibitor | Dorsomorphin and its derivatives (CDN-193189, CDN-212854) | By inhibiting ALK2/3 mediated Smad phosphorylation, the extent of ossification and functional disorders are reduced. | 1.The BMP pathway also plays a key regulatory role in OPLL.  2.Multiple inhibition strategies targeting the BMP/Smad axis, especially ALK2/3, may offer promising non-surgical treatment options for OPLL. | It has certain toxicity and side effects, and its clinical transformation is limited. | (Tanaka et al., 2001; Williams et al., 2018; Yonemori et al., 1997; Yu et al., 2008) |
| RARγ agonist | Palovarotene | It inhibits osteogenic differentiation, angiogenesis and inflammatory response through pathways such as PI3K-Akt, PPAR, P53, VEGF, Smad and NF-κB, thereby slowing down the process of HO. | OPLL and HO share certain commonalities in osteogenic mechanisms, and the application potential of Palovarotene in OPLL should be further developed. | It has shown potential value in HO studies, especially in significantly improving FOP, but there is also a risk of premature closure of the growth plate | (Hsiao et al., 2025; Huang, Lin, et al., 2022; Huang, Liu, et al., 2022) |
| Wnt pathway inhibitor | Dkk1 | By blocking the Wnt/β-catenin signaling pathway, osteogenic transformation of ligament cells is inhibited. | 1. The abnormal activation of Wnt/β-catenin signaling is involved in the pathological process of OPLL.  2. The expression of the endogenous inhibitory factor Dkk1 of Wnt is decreased in the serum of patients with OPLL. | It may affect bone metabolism throughout the body | (Dong et al., 2020; Niu et al., 2017; Shi et al., 2016) |
| HIF-1α pathway inhibitor | PX-478、imatinib、Apigenin、Rapamycin | Effectively inhibit the activity of HIF-1α and its downstream osteogenic and angiogenic signaling pathways, thereby slowing down HO. | After activation, HIF-1α promotes angiogenesis and bone formation by regulating genes such as BMP, VEGF and NRP-1, and accelerates HO. | It has a remarkable effect in the FOP model and may be transferred to the OPLL study for application. | (Huang et al., 2020; Qureshi et al., 2017; Wang et al., 2016) |
| TNAP inhibitor +PPi | Levamisole+ Exogenous PPi | Targeting pyrophosphate metabolism, it significantly inhibits the pathological process of the OPLL model. | PPi is a natural mineralization inhibitor in the body, but its exogenous form is easily hydrolyzed by TNAP. | There are no side effects such as osteoporosis. | (Hiratsuka et al., 2018) |
| sEVs-miRNAs | miR-320e | miR-320e inhibits TAK1, disrupts the local bone metabolic balance and promotes ectopic bone formation. | 1. Ligament cells in OPLL patients can secrete sEVs rich in miR-320e.  2. Injection of sEVs derived from OPLL can accelerate the formation of spinal canal osteophytes. Blocking miR-320e or inhibiting the secretion of sEV can significantly alleviate the lesion. | The miR-320e/TAK1 axis is a new target for the treatment of OPLL. | (Xu et al., 2022) |
| Specific targeted regulation of miRNAs | miR-218 | Target and inhibit the expression of osteogenic transcription factors Runx2 and COL1A1, and negatively regulate the OPLL process. | The expression level of miR-218 in ligament cells of OPLL patients was significantly lower than that of non-OPLL patients. | — | (Gay et al., 2014; Liu et al., 2018; Xu et al., 2016) |
|  | miR-140-5p | Target IGF1R, inhibit its downstream IRS1/PI3K/Akt/mTOR signaling pathway, and suppress the osteogenic differentiation of hMSCs. | miR-140-5p is down-regulated in exosomes of OPLL-derived cells. | — | (Tang et al., 2022) |
| H2RAs | famotidine | The expressions of Runx2, OCN, etc. were regulated through the HRH2-cAMP/PKA/CREB pathway to reduce the ALP activity and the formation of mineralized nodules in OPL-Mscs. | HRH2 expression is upregulated in MSCs derived from OPLL patients and participates in the regulatory process of osteogenic differentiation of cells. | It has wide clinical applications. It has the potential to inhibit the progression of OPLL and provides a realistic and feasible direction for drug redevelopment. | (Kim et al., 2013; Liu et al., 2017; Wellner-Kienitz et al., 2003; Zhang et al., 2015) |

Dkk1: dickkopf-related protein 1; FOP: fibrodysplasia ossificans progressiva; ALK: anaplastic lymphoma kinase; TAK1: transforming growth factor-activated kinase 1; IRSs: insulin receptor substrate; IGF1R: insulin-like growth factor 1 receptor; hMSCs: human mesenchymal stem cells; HRH2: histamine receptor H2; cAMP: cyclic AMP; PKA: protein kinase A; CREB: cAMP-response element binding protein; OPLL-MSCs: MSCs from OPLL patients.

**Supplementary Table 2. OPLL intervention strategies targeting angiogenesis**

| **Measures** | **Target** | **Mechanism** | **References** |
| --- | --- | --- | --- |
| sorafenib | The LOXL2/HIF-1α/VEGF signaling axis | 1. Inhibit LoxL2-mediated endothelial-like differentiation of ligament cells and reduce the formation of capillary-like structures;  2. Delay BMP-induced ectopic bone formation (BIO model) and ENPP1-deficient deficient OPLL-like lesions (LSO model);  3. Block VEGFR and PDGFR, disrupt the coupling of angiogenesis and ossification, inhibit the osteogenic differentiation of ligament cells, and reduce H-type angiogenesis. | (Bae et al., 2018; Baker et al., 2013; Coral et al., 2013; Wang et al., 2024) |
| Cyclo(RGDyK) | Integrin αVβ3/FAK/ERK signaling axis | 1. Block αVβ3, down-regulate the phosphorylation levels of FAK and ERK1/2, inhibit the expression of Runx2, and weaken the osteogenic differentiation of LFs;  2. Reduce the levels of p-VEGFR2, p-FAK and p-ERK1/2, and inhibit the migration of ECs and angiogenesis. | (Geng et al., 2024) |
| ANGPT inhibition or ANGPT knockdown | ANGPT/Notch signal axis | 1. By interfering with the stabilizing effect of the ANGPT/Tie2 axis, the adhesion of ECs, vascular permeability and the growth pattern of new blood vessels are altered.  2. By inhibiting the expression of downstream bone formation markers through Tie2/Notch2, ossification can be suppressed. | (Yang et al., 2018) |
| Stat3 activator or overexpression of miR-135b | IL-6/Stat3/miR-135b signal axis | Inhibit the expression of BMPER, disrupt the vascular-ossification coupling mechanism, and suppress osteogenic differentiation. | (Ji et al., 2023; Xiao et al., 2018) |
| TGF-β inhibition | TGF-β/ALK/Smad signal axis | 1. Interfere with the migration, proliferation and pericytes recruitment of ECs, and inhibit the formation of new blood vessels; And indirectly inhibit the activation of Smad2/3, and suppress the ossification process;  2. Interfere with EndMT;  3. Slow down the PDGF-BB signal released after bone resorption, inhibit H-type angiogenesis, and slow down the osteogenic process of OPLL;  4. Inhibit the inflammatory microenvironment;  5. Interfere with the communication of cells related to angiogenesis and thereby affect the remodeling of the bone vascular system. | (Goumans et al., 2002; Li et al., 2025; Ma et al., 2020; X. Wang et al., 2018; Zhang et al., 2025; Zhang et al., 2020) |
| *ACE* targeted therapy | *ACE* D/D-Ang II-VEGF signal axis | Inhibit angiogenesis and the OPLL process. | (Brasier, 2010; Carbajo-Lozoya et al., 2012; Kim et al., 2014; Rigat et al., 1990; Tamarat et al., 2002) |
| verteporfin | YAP/β-catenin signal axis | Inhibiting the expression of osteogenesis-related genes (*Runx2*, *COL1A1*, *OCN*) and angiogenic factors (VEGFA, CD31, ANGPT1) can effectively slow down the heterotopic ossification of tendons or ligaments, and may also inhibit OPLL. | (Luo et al., 2023) |

Cyclo(RGDyK): [Cyclic Arg-Gly-Asp-D-Tyr-Lys](https://www.chembk.com/cn/chem/Cyclic Arg-Gly-Asp-D-Tyr-Lys).
